# A review of 413 salivary gland tumours in the head and neck region

**DOI:** 10.4317/jced.51143

**Published:** 2013-12-01

**Authors:** Ahmed O. Lawal, Akinyele O. Adisa, Bamidele Kolude, Bukola F. Adeyemi, Mofoluwaso A. Olajide

**Affiliations:** 1 FMCDS, Lecturer/Consultant. Department of Oral Pathology, College of Medicine, University of Ibadan, Nigeria; 2 FWACS, Lecturer/Consultant. Department of Oral Pathology, College of Medicine, University of Ibadan, Nigeria; 3 BDS, Senior Registrar. Department of Oral Pathology, University College Hospital, Ibadan, Nigeria

## Abstract

Objectives: Salivary gland tumours (SGTs) are a group of heterogeneous lesions with complex clinico-pathological characteristics and distinct biological behaviours. Previous studies have reported geographic variations in site distribution, incidence and histological types of SGTs. The aim of this study was to describe the demography of SGTs seen at a tertiary health centre and compare findings with previous studies. Study design: Data on SGTs from archives of the Department of Oral Pathology and the Department of Pathology, University College Hospital Ibadan were retrieved. Information about histological types, age, sex and location were analyzed using SPSS for Window (version 20.0; SPSS Inc. Chicago, IL). Reactive and tumor-like lesions such as sialometaplasia, benign lymphoepithelial lesion, lymphoepithelial cyst, mucocele, mucous extravasation phenomenon, ranula, and sialosis were excluded from the study. Results: 413 SGTs consisting of 221 (53.5%) malignant and 192 (46.5%) benign lesions were seen. SGTs occurred more in females (50.6%) than males (49.4%) with a mean age of 43.7 (±16.9) years and peak age in the fifth decade of life. The parotid with 171 (41.4%) cases was the commonest site, followed by palate with 89 (21.5%) cases, while only 7(1.7%) cases were seen in sublingual gland. Pleomorphic adenoma with 169 (40.9%) was the most frequent SGT followed by adenoid cystic carcinoma with 93 (22.5%) cases which also was the most frequent malignant SGT while only 3 (0.7%) cases of Warthin’s tumour were seen. Conclusion: This report is one of few that showed a higher occurrence of malignant SGTs compared to their benign counterparts. The findings were essentially similar to findings in Africa but showed SGTs to be more common in females. The reason(s) for high occurrence of malignant SGTs in minor salivary glands and the rarity of Warthins tumour in this study and other African series compared to those from America needs further investigation.

** Key words:**Salivary gland tumours, parotid gland, pleomorphic adenoma, adenoid cystic carcinoma, warthin’s tumour.

## Introduction

Salivary gland tumours (SGTs) are a group of heterogeneous lesions with complex clinico-pathological charac-teristics and distinct biological behaviours (1). SGTs are rare and represent less than 3% of all head and neck tumours (2) but they are clinically significant because of their histological and behavioural diversity. Considerable challenges with management may also arise because of their proximity to important head / neck structures (3). Due to the diversity of SGTs, histological classification was fraught with discrepancies. These discrepancies have somewhat been resolved by the World Health Organization’s (WHO) Histological Classification of SGTs established in 1971, first revised in 1991 and then 2005, thus establishing a reliable standard for diagnosis and study (4).

Reports from previous studies show a wide geographic variation in the site distribution, incidence and histological types of SGTs, however, most studies show pleomorphic adenoma to be the commonest SGT (1,4-7). Although, the most common malignant SGT varies from one study to another, either adenoid cystic carcinoma (ACC) or mucoepidermoid carcinoma (MEC) are usually reported as the most common malignant SGTs (1,5-6). The parotid gland is the most frequently affected site for SGTs, with the submandibular gland and palate being other commonly affected sites while the sublingual gland is usually the least affected site. Also, reports have shown varying ratios of malignant and benign SGTs in the different sites, as the parotid gland tend to have a higher percentage of benign lesions while the sublingual and the minor salivary glands seem to have a higher percentage of malignant SGTs (4,6).

Many factors have been implicated in the development of SGTs, among which is low dose radiation and ultraviolet radiation. Other factors implicated include; occupational exposure to wood and wood dust, alcohol and hair dyes in women and Epstein-Barr virus (6). More so, an increased incidence of salivary gland neoplasms has been reported among patients with breast cancer (6). Though many studies have examined the demography of SGTs, most of them did not include the extra-oral minor salivary glands. The aim of this study was to describe the demography of SGTs of the head and neck region seen at a tertiary health centre based on WHO 2005 classification of SGTs and compare findings with other studies.

## Material and Methods

Archival data of the Department of Oral Pathology and the Department of Pathology, University College Hospital Ibadan spanning 19 years were retrieved. Information about SGTs histology, location, patients age and gender were analysed using SPSS for Windows (version 20.0; SPSS Inc. Chicago, IL). All cases were categorised according to the 2005 WHO Organization histological classification. Reactive and tumor-like lesions like sialometaplasia, sialosis, benign lymphoepithelial lesion, lymphoepithelial cyst, mucous retention and mucous extravasation phenomena were excluded from the study. Ethical approval for the study was obtained from the Oyo State Research Ethical Review Committee (AD 13/479/435).

## Results

A total of 413 SGTs; consisting of 221 (53.5%) malignant and 192 (46.5%) benign lesions were seen within the study period.  Table 1 shows a summary of the demographic distribution of SGTs. Generally, SGTs were slightly more common in females (50.6%) than males (49.4%). The mean age of occurrence was 43.7 (±16.9) years with peak age in the fifth decade of life.  Table 2 shows the various histologic types of SGTs seen. Pleomorphic adenoma (PA) was the most frequently seen SGT with 169 cases representing 40.9% of all SGTs and 88.0% of benign SGTs. The mean age of occurrence for malignant SGTs was 47.9 (±17.0) years while the mean age was 38.2 (±15.5) years in benign SGTs. There was a statistically significant difference in the mean ages of malignant SGTs and benign SGTs (p=0.00). Malignant SGTs occurred more in males than in females with a male: female ratio of 1.2:1 while benign SGTs occurred more in females than males (male: female ratio= 1:1.3).

The parotid with 171 (41.4%) cases was the commonest site, followed by submandibular and sublingual glands with 49 (11.9%) and 7 (1.7%) cases respectively. The palate with 89 (21.5%) cases was the commonest site of occurrence in the minor salivary glands followed by buccal mucosa with 17 (4.1) cases.  Table 3 shows the site distribution of the most commonly seen SGTs.

**Table 1:**
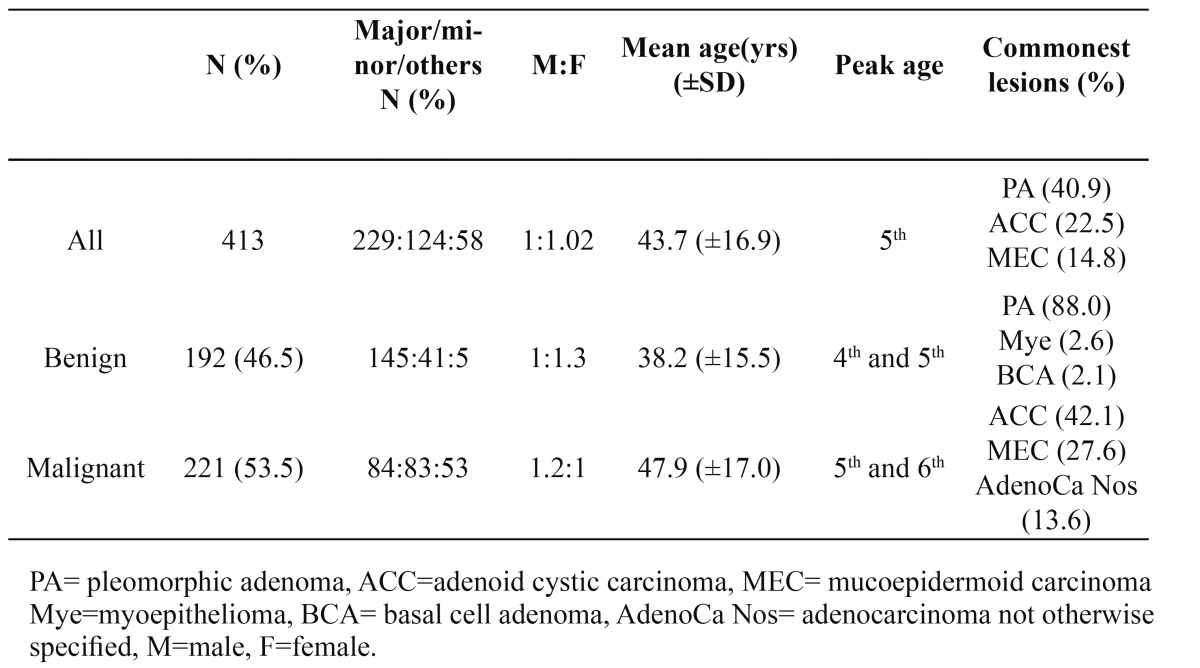
Overview of the Demography of SGTs.

**Table 2:**
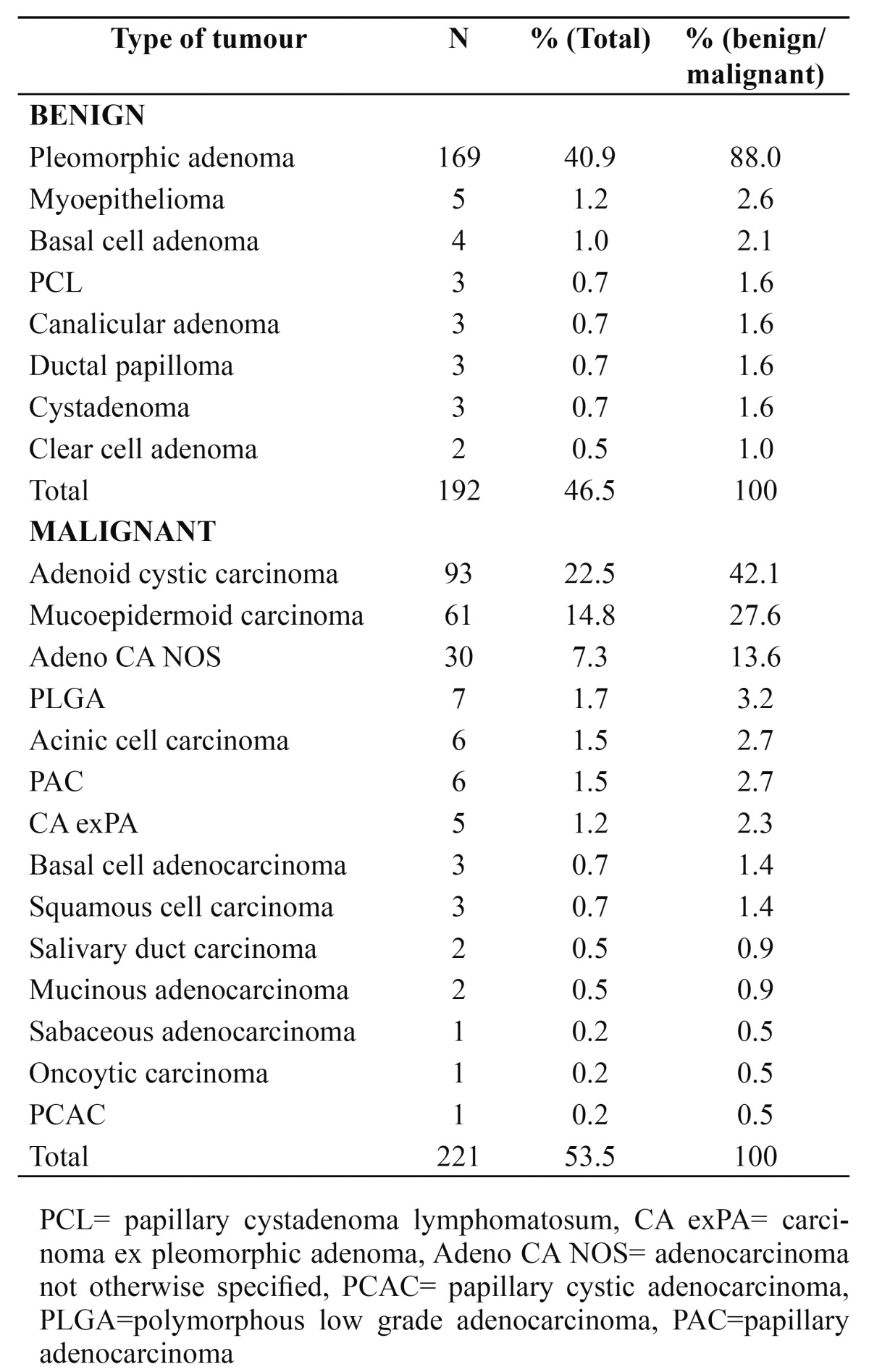
Histologic types of Salivary gland tumours.

**Table 3:**
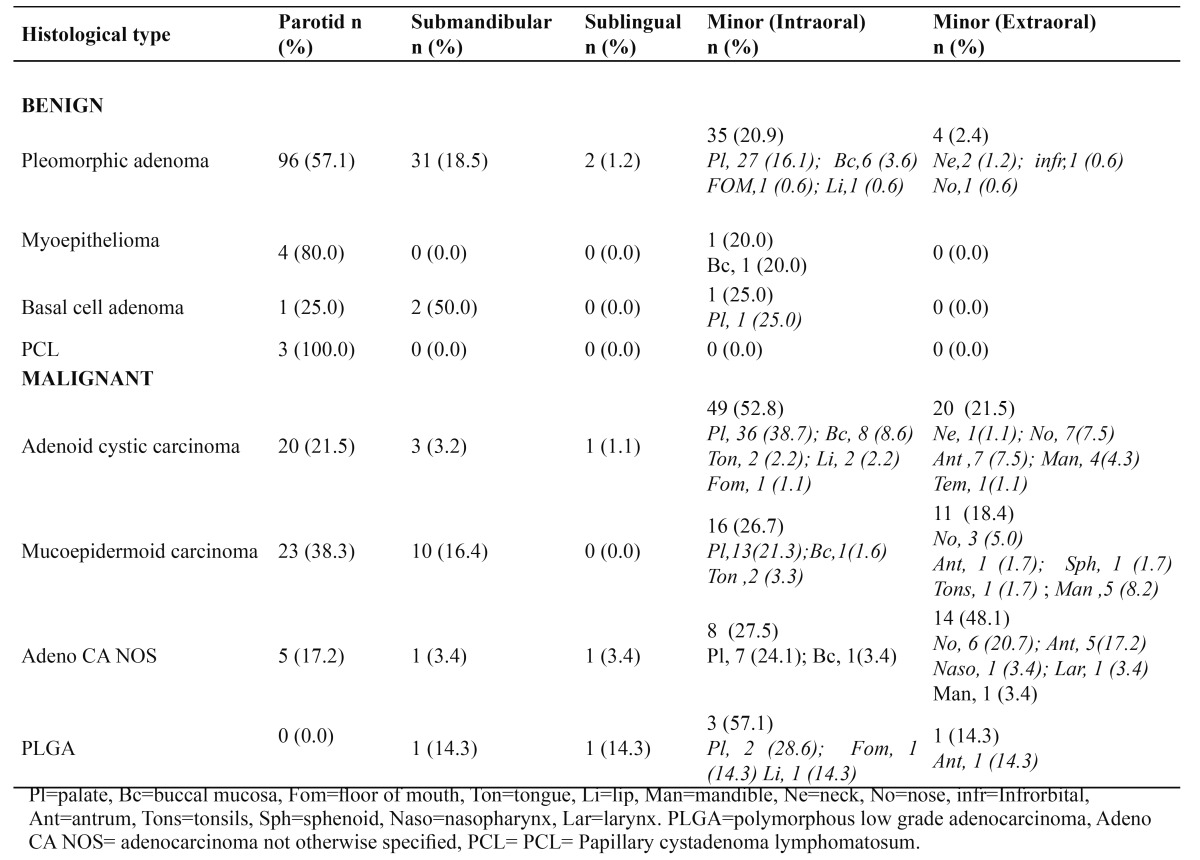
Distribution of the most common types of SGTs according to site.

## Discussion

This study reviewed 413 cases of SGTs and showed that malignant SGTs were more common than benign SGTs representing 53.5% of all cases. This is at variance with previous studies that reported benign SGTs as commoner than malignant with varying values 78.3% (1), 67.5% (2), 59.7% (4), 70.0% (6) and 55.7% (8). The reason for this uncommon finding may be due to the fact that our centre is a referral tertiary centre and the ?more severe cases’ of SGTs, which are more likely to be malignant, will be referred to our centre. The ? less severe cases’ which are more likely to be benign (especially the intraoral lesions) are more likely to be treated at primary and secondary health facilities. There is also the possibility that malignant SGTs are more prevalent than benign SGTs in our study environment due to yet to be determined genetic or environment factors. Another study from south-west Nigeria by Ladeinde et al. (9) also reported a higher prevalence of malignant SGTs in their study.

The parotid gland with 171 (41.4%) cases was the commonest site followed by minor salivary glands (30%), the submandibular gland (11.9%) and the sublingual gland (1.7%). This was in conformity with all large series which showed parotid gland to be the commonest site of occurrence (2). Otoh et al. (8) and Kolude et al. (10) in previous studies from Nigeria reported that 45.6% and 46.5% respectively of SGTs occurred in the parotid gland. However, this was at variance with the finding of Ladeinde et al. (9) which showed minor salivary gland as the most common site of occurrence of SGTs. Oliveira et al. (1) and Ito et al. (2) both from Brazil got higher percentages of 67.7% and 69.5% respectively. Chidzonga et al. (7) suggested that racial variations may exist in the clinico-pathologic distribution of SGTs with African cases having less proportion of SGTs in parotid gland but more proportion occurring in the submandibular and minor salivary glands when compared with white populations in Europe and America ( Table 4). This suggestion seems to be supported by this study and may be due to the rarity of Warthin’s tumour (which almost exclusively occurs in the parotid) in Africans when compared with Caucasians ( Table 4). This study found only 7 (1.7%) of SGTs in the sublingual gland and this supports various reports about the rarity of SGTs in the sublingual gland as reported by previous authors, though some authors found no SGTs in the sublingual gland (1,2,7,11). Satko et al. (5), on the other hand, reported that sublingual tumours represented 3.2% of all SGTs in their study.

**Table 4:**
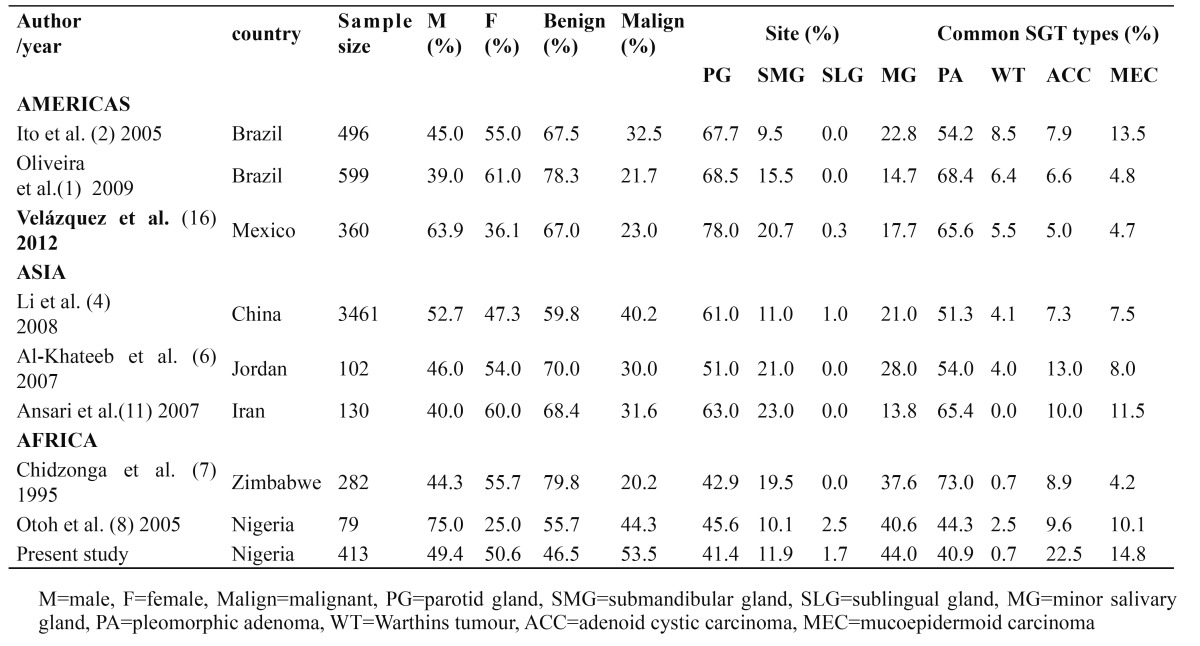
Comparison of studies on SGTs.

PA to be the commonest SGT in this study accounting for 40.9% of all SGTs and 88.0% of benign SGTs. Previous studies have all shown PA to be commonest SGT (1,4-6,12,13). The incidence of PA has been reported to range from between 40.8% to 70.4% (14). ACC was the second most common SGT and the most common malignant SGT which was corroborated by Chidzonga et al. (7) from Zimbawe, Ostman et al. (15) from Sweden, Satko et al. (5) from Brastislavia and Otoh et al. (8) from Northeastern Nigeria. In contrast, Ansari (11) from Iran, Ito et al. (2) from Brazil, AlKhateeb et al. (6) from Jordan and Kolude et al. (10) from Nigeria found MEC to be the commonest malignant SGT in their studies.

Only 3 (0.7%) cases of Warthin’s tumour were seen in this report. There appears to be a geographical variation in the prevalence of Warthin’s tumour as reports from Brazil (2), Mexico (16), Slovakia (5), and China (4) show prevalence of 4.4% to 10.5% while middle east (6,11) and African studies (7,8) indicate that it is a rare SGT (0 - 2.5% of SGTs). This disparity may be due to the strong association of Warthin’s tumour with tobacco use and the rarity of the tumour in Africans and Arabs may be explained by the relatively less frequency of tobacco use in these regions.

In the present study, the age range was 6-98 years, the mean age of occurrence was 43.7 years and the peak age incidence was in the 5th decade of life which was in conformity with previous studies (4,11,17). The mean age for malignant SGTs (47.8 years) was significantly higher than mean age for benign SGTs (38.8years) and peak age was in the fifth and sixth decades for malignant SGTs while it was in the third and fourth decades for benign SGTs. These findings in our study are comparable to those of Li et al. (4) in China. In contrast, Oliveira et al. (1) from a study in Brazil, reported median ages of 55 and 43 years for malignant and benign SGTS respectively while their peak age were in the seventh and fourth decades respectively.

Previous studies from Africa (7,8,10) showed that SGTs were more common in men than in women, which is at variance with our finding of 50.6% in females and 49.4% in males. However, our finding was in conformity with many studies from other climes which showed either almost equal sex distribution (4) or a slight female preponderance (1,5). Also, our finding of a slight female preponderance for benign SGTs and a slight male preponderance for malignant SGTs was in conformity with previous studies from China and Brazil (1,4).

To the best of the author’s knowledge, this report on SGTs is the largest series from Africa and one of few to show a higher prevalence of malignant SGTs. Our findings were essentially similar in most respects to reports from Africa but showed SGTs to be more common in females. The reason(s) for the relatively high occurrence of SGT in minor salivary glands and the rarity of Warthins tumour in this study and other African series compared to those from America need further investigation.
